# Could Hyaluronic Acid Be Considered as a Senomorphic Agent in Knee Osteoarthritis? A Systematic Review

**DOI:** 10.3390/biomedicines11102858

**Published:** 2023-10-22

**Authors:** Andrea Bernetti, Francesco Agostini, Marco Paoloni, Maria Vittoria Raele, Giacomo Farì, Marisa Megna, Massimiliano Mangone

**Affiliations:** 1 Department of Biological and Environmental Sciences and Technologies (DiSTeBA), Università del Salento, 73100 Lecce, Italy; giacomo.fari@unisalento.it; 2 Department of Anatomical and Histological Sciences, Legal Medicine and Orthopedics, Sapienza University, 00189 Rome, Italy; francesco.agostini@uniroma1.it (F.A.); marco.paoloni@uniroma1.it (M.P.); massimiliano.mangone@uniroma1.it (M.M.); 3 Department of Translational Biomedicine and Neuroscience (DiBraiN), Aldo Moro University, 70121 Bari, Italy; maryvi.92@hotmail.i (M.V.R.); marisa.megna@uniba.it (M.M.)

**Keywords:** knee osteoarthritis, hyaluronic acid, injection, intraarticular drug therapy, viscosupplementation, senomorphic

## Abstract

Background: Knee osteoarthritis (KOA) is one of the most common causes of disability in elderly patients and tends to be a major burden on social and health care spending. Despite its severe socioeconomic impact, KOA remains, to date, an incurable disease. Due to its proper characteristics, KOA represents a favorable disease model for experimenting with senotherapeutics, a group of treatments that counteract the development of age-related disorders and chronic diseases. In recent years, the use of intra-articular hyaluronic acid (IAHA) in the treatment of diseases related to the wear and tear of the articular cartilage has been gaining popularity. Given its ability in joint lubrification, shock absorption, and cell signaling, our aim is to investigate, through the existing scientific literature, its potential role as a senomorphic agent, emphasizing its crucial function in KOA patients. Indeed, senomorphics are a particular group of senotherapeutics capable of modulating the functions and morphology of senescent cells to those of young cells or delaying the progression of young cells to senescent cells in tissues. Methods: A search in the scientific literature (PubMed, Cochrane Library, and Google Scholar) was carried out from 2019 to 2023, thus the last 5 years. Results: One hundred thirty-eight articles were found concerning the role of hyaluronic acid injections in KOA patients. In these studies, its therapeutic efficacy, its anti-inflammatory properties, and its low risk of side effects emerged. Conclusion: IAHA injections are a valuable treatment option for KOA while they can provide pain relief, improve joint function, and slow the progression of joint degeneration. The inhibitory effect of HA on MMP13 and its action as a senomorphic agent suggests that it may have additional benefits beyond its lubricating and shock-absorbing properties. In order to clarify its mechanisms of action and to optimize its clinical use, further studies are definitely needed.

## 1. Introduction

Osteoarthritis (OA) is a chronic degenerative disease that affects the joints involving all the tissues that are part of them [1]. It is characterized by the progressive loss of articular cartilage, synovitis, osteophyte formation, subchondral bone sclerosis, and a progression of inflammatory and fibrotic processes that lead to degeneration of the joint itself [2]. OA is the most prevalent type of arthritis, affecting approximately 250 million individuals worldwide, and its prevalence and incidence are expected to rise in the coming years, especially due to an increase in the average age of the world’s population. It can involve many different joints, but the knee is most frequently affected, followed by the hands, the hip, and the spine [3]. Knee osteoarthritis (KOA), also known as gonarthrosis, consists of a heterogenous peripheral joint disorder characterized by a complex and multifactorial nature [4] and with multiple risk factors that can be divided into modifiable, such as obesity or a smoking habit, and not modifiable ones, such as age, female sex, and genetic predisposition [5]. It represents one of the most common causes of disability in elderly patients [6], leading to joint deformity, pain, and functional loss. Moreover, it is now well demonstrated in the existing literature that this condition is increasing even in people aged from 45 to 70 years [7]. Being associated with functional limitation and pain, KOA has a negative impact on social connectedness and psychological well-being, reducing patients’ quality of life (QoL) [8], increasing the phenomenon of absenteeism from work, and clearly burdening the health care costs, mainly affecting the population of industrialized countries [9]. For this reason, its early symptoms should be immediately detected with the aim of promptly intervening with a proper approach consisting of structured education, targeted therapeutic exercises, and lifestyle adjustment, trying to limit the now-known modifiable risk factors such as obesity and smoking habit [10]. KOA, unfortunately, remains, nowadays, the greatest challenge in the field of OA, with a high morbidity load and unavailable definitive solutions in terms of treatments [11].

Due to its proper characteristics, KOA represents a favorable disease model for experimenting with senotherapeutics, a group of treatments which counteract the development of age-related disorders and chronic diseases. In fact, like all the biological tissues of the human body, joints also are destined for senescence and decay, and the increasing number of cartilage and bone senescent cells related to aging is directly linked with OA pathogenesis [12]. Moreover, the knee joint specifically, tends to be the most likely to senescence due to the need to support body weight and, therefore, lends itself more to a local treatment with senotherapeutics [13].

Senescence is a process in which cells enter a state of irreversible growth arrest in response to various stressors, such as DNA damage or oxidative stress. Senescent cells can accumulate in tissues with age and contribute to chronic inflammation and tissue degeneration. Aging and accumulation of senescent cells with age contributes to tissue or organismal aging, causing many specific age-related pathologies based on these involutional processes [14,15]. The two strategies available to combat senescence at the tissue level are the senolytic strategy, which induces the apoptosis of aged and damaged cells, and the senomorphic strategy, which instead controls the production of the senescence-associated secretory phenotype (SASP), or of all those substances, such as cytokines, metalloproteinases, involved in the damage linked to cellular aging.

One of the major challenges in treating KOA is to slow down or rather reverse the process; however, nowadays, these goals have not been fully achieved. In fact, although it is possible to at least partially slow down the arthritic process, cartilage regeneration is still an unattainable goal. Conservative treatments, such as nonsteroidal anti-inflammatory drugs (NSAIDs), nutraceuticals, physical therapy, patient education for a proper lifestyle, and weight loss, may relieve symptoms and improve articular function, but fail to halt disease progression [7].

In recent years, the use of hyaluronic acid (HA) in the treatment of diseases related to the wear and tear of the articular cartilage has been gaining popularity [16]. Indeed, it is a glycosaminoglycan that is abundantly present in the synovial fluid, cartilage, and other connective tissues [17]. It is endogenously produced by the human body and has unique physicochemical and biological properties, exhibiting desirable biocompatibility and biodegradability [18]. HA plays a crucial role in joint lubrication, shock absorption, and cell signaling and, even if it was demonstrated that exogenous HA is unable to restore or replace the properties and activities of endogenous HA, it can still provide satisfactory results in different fields and especially the OAs one. Therefore, considering the progressive and widespread clinical use of this therapeutic procedure, we aimed to perform an innovative systematic review of the existing literature concerning its potential role as a serotherapeutic, and precisely, a senomorphic agent, in the treatment of KOA.

## 2. Materials and Methods

This systematic review was conducted following the PRISMA guidelines (Preferred Reporting Items for Systematic Reviews and Meta-Analyses) to ensure transparency and rigor in the review process. The systematic review intended to study all the existing literature regarding the potential role of intra-articular hyaluronic acid (IAHA) injections in the treatment of KOA disease. In comparison to the studies already reported in the literature, this systematic review aims to emphasize all the properties and characteristics of IA that might classify it as a senomorphic agent, capable of modulating functions and morphology of senescent cells to those of young cells and delaying the progression of young cells to senescent cells in tissues. The literature was searched for on PubMed, Cochrane Library, and Google Scholar, considering articles published between 2019 and 2023, thus of the last 5 years. The search terms were “hyaluronic acid injection” and “hyaluronic acid infiltration” combined with “knee osteoarthritis”, “osteoarthritis of the knee” and “gonarthrosis”. The exclusion criteria were as follows: full text not available, articles not in English language, articles in which hyaluronic acid was combined with other therapies as the aim was to highlight its own potential alone, protocols and in vitro studies or in animal models to focus attention on achievements in human being.

The authors independently performed the search and removed duplicate records. Opinions expressed in this systematic review are also based on personal experience of writing, editing, and commenting on review articles.

## 3. Results

**Figure 1:**
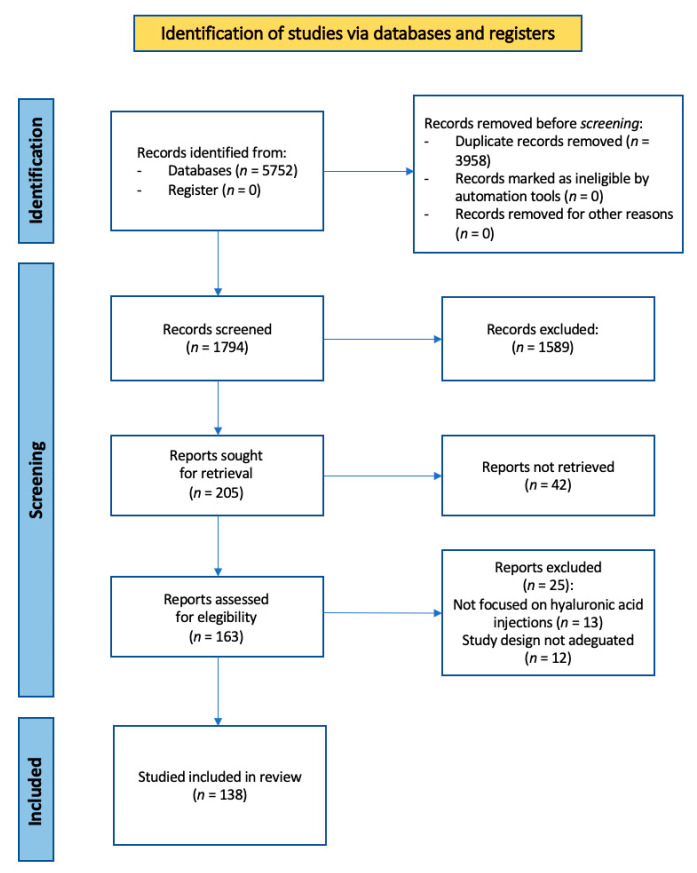
PRISMA 2020 flow diagram.

After the careful elimination of duplicates and based on the previously mentioned exclusion criteria, the search resulted in a total of 138 articles that can be divided into two main categories precisely, clinical trials and reviews. (Figure 1 PRISMA flow chart).

The recurring theme of these articles has been to compare the efficacy of IAHA in KOA patients with other treatments of established efficacy, such as platelet-rich plasma (PRP), plasma rich in growth factors (PRGF), ozone, and corticosteroids. In this regard, in 2021, it was demonstrated that a molecular weight between 500 and 730 k Daltons HA buffered in physiological sodium chloride, PRP, and PRGF injections were superior to ozone in improving the algic symptoms in KOA patients for a longer period [19]. Although the superiority of PRP over HA treatment in this category of patients is now approved and established [20,21], in a group of obese patients, the difference between these two products turned out to be almost zero in the short-term, while it increased in favor of PRP at 6-, and especially 12-months, after the end of the treatment [22]. Recently, the prior Osteoarthritis Research Society International (OARSI) guidelines were updated and expanded by the development of patients’ personalized treatment recommendations for individuals with knee, hip, and polyarticular OA [23]. Intra-articular corticosteroids, IAHA, and aquatic exercise resulted in Level 1B/Level 2 treatments for KOA patients (not for hip or polyarticular OA), dependent upon comorbidity status, following topical non-steroidal anti-inflammatory drugs (NSAIDs), which were considered Level 1A, and being at the same level of COX-2 inhibitors for individuals with gastrointestinal comorbidities (Level 1B) and NSAIDs with proton pump inhibitors (Level 2). Findings regarding the differences between the use of intra-articular corticosteroids and IAHA in KOA patients are conflictual. Also, HA does not seem to have a significantly higher number of side effects when compared to saline or corticosteroid injections and provides better medium-term control of symptoms in patients with mild to moderate KOA [24]. It was demonstrated that KOA patients treated with IAHA had lower total medical care costs, fewer side effects, and lower use of opioids and analgesics prescriptions compared to KOA patients treated with intra-articular corticosteroids [25]. In addition, while the results at 3 months depose for a better effect achieved by corticosteroid injections in patients with gonarthrosis, it appears that at 6 months, the effect of corticosteroids fades almost completely in favor of HA, which, instead, keeps the knee joint pain free in many patients affected [26]. It must be noted that one of the limits of these studies is that researchers collected data, including different types of HA, without specifying their molecular weights and, therefore, increasing the risk of bias.

Moreover, among these studies, the HA anti-inflammatory potential and its specific action in slowing down the articular cartilage degeneration process were reported. A systematic review focused on the general anti-inflammatory effects of HA in KOA, mediated through the receptor-binding relationship with cluster determinant 44 (CD44), toll-like receptor 2 (TLR-2) and 4 (TLR-4), intercellular adhesion molecule-1 (ICAM-1), and layilin (LAYN) cell surface receptors [27]. This review pointed out that a higher molecular weight HA (HMWHA) promotes anti-inflammatory responses, while short HA oligosaccharides produce inflammatory reactions. Mechanistically, HA demonstrated its involvement in attenuating phagocytosis, as well as decreasing prostaglandin, fibronectin, and cyclic adenosine monophosphate levels. A hypothesis is also that HA could prevent the release of arachidonic acid, block nociceptors, and decrease the bradykinin and substance P formation [28]. The anti-inflammatory, growth-modulating properties of certain cells and their differentiation into chondrocytes of HA have been demonstrated in a study that focused on investigating a therapeutic treatment capable of regenerating the meniscus in 18 patients who underwent total knee arthroplasty [29]. Indeed, it appears that the binding of HA to its main receptor, CD44, activates the phosphoinositide 3-kinase (PI3K) and mitogen-activated protein kinase (MAPK) pathways, generating a proliferation and differentiation of cartilage cells. Of considerable importance turns out to be a recent multi-center randomized controlled study, which deals with the different changes in the biomarker C-terminal telopeptides of type II collagen (CTX-II) following the intra-articular injection of high molecular weight hyaluronic acid and oral non-steroidal anti-inflammatory drugs in patients affected by KOA [30]. The findings of this study disclosed that both treatments improve symptoms in KOA patients but through different modes of action as follows: specifically, while intra-articular hyaluronic acid stimulates cartilage/bone interface extracellular matrix type II collagen turnover, NSAIDs seem to reduce such turnover. Although further studies are crucially needed, this multi-center randomized controlled study could be a starting point to explain the role of HA in slowing down the articular cartilage degeneration process.

Finally, HA safety and practicality emerged in numerous literature reviews. As shown in the results from a meta-analysis published in 2019 [31], IAHA seems not to be associated with any safety issue in the management of OA in general, and KOA in particular. Moreover, in a systematic review written by Chavda S et al. [32] in 2022 it was demonstrated that IAHA injections are safe and effective, and the most common side effects are just some minor effects, such as local pain and swelling, which usually appear a few hours after the HA injection and generally tend to spontaneously resolve within few days with no serious consequences. Severe allergic reactions are extremely rare, and their probability increases when HA is combined with other KOA treatments. For this reason, the combination of drugs should always be monitored.

## 4. Discussion

In KOA, the concentration and molecular weight of HA in the synovial fluid decrease, which impairs its lubricating and anti-inflammatory functions [12]. The IAHA injection aims to restore the HA concentration and rheological properties in the joint space, which may reduce pain, improve joint function, and slow down the disease progression [19]. In addition to its physical properties, HA has been shown to exert chondroprotective effects by modulating various cellular and molecular pathways [33]. Indeed, it can stimulate chondrocyte proliferation, differentiation, and extracellular matrix (ECM) synthesis, which are essential for maintaining cartilage integrity and inhibiting chondrocyte apoptosis, which is increased in KOA and contributes to cartilage degradation [34]. Moreover, it is capable of upregulating the expression of type II collagen and aggrecan, the main components of cartilage ECM, and, on the other hand, downregulating the expression of matrix metalloproteinases (MMPs) [34]. Several in vitro and in vivo studies have demonstrated the chondroprotective effects of HA in OA, although the precise mechanisms of the action are not fully understood [33,34,35]. Based on its above-mentioned properties, an IAHA injection can be considered as a promising therapy for KOA. The osteoarthritic knee is certainly the most investigated joint for the effectiveness of all the most important OA therapies, especially for this mini-invasive treatment [12]. Although in recent years regenerative medicine has extensively experimented with new drugs such as PRP and mesenchymal cells with very promising results, HA remains a solid and scientifically well-validated pillar of infiltrative KOA therapy [36]. In fact, in patients affected by KOA, the concentration and molecular weight of HA in the synovial fluid are reduced, and it contributes to joint degeneration and inflammation [12]. By injecting exogenous HA into the joint, the concentration and molecular weight of HA can be increased, leading to improved joint function, reduced pain, and allowing affected patients a better QoL, decreasing the pathology psychological impact and the health care costs [8].

Several clinical trials have demonstrated the effectiveness of intra-articular HA injections for KOA. A meta-analysis of 29 randomized controlled trials showed that HA injections were more effective than placebo injections for reducing pain and improving function in patients with KOA [37]. It must be said that the methodological quality of the meta-analyses dedicated to infiltrative practices in the KOA, as well as in other joints, does not always guarantee solid levels of evidence. The differences in the procedures and protocols adopted, as well as the evaluation scales that are often too subjective, put the results at risk of strong bias. Indeed, the literature highlights the need to detail osteoarthritis characterization, including subgroup analyses and osteoarthrosis severity stratification, and standardize the procedures and the assessment scales for IA injections in forthcoming research in order to obtain valid guidelines and the lowest possible risk of bias [38]. However, this evidence exists for KOA injections. Richette et al. [39] collected clinical data obtained from IA placebo-controlled trials with a low risk of bias only to reach the highest level of evidence, and they demonstrated a moderate but real effect of IAHA on pain in patients with KOA based on an ES of 0.20 (95% CI 0.12, 0.29) for pain. Petrella et al. [40] retrospectively investigated the effectiveness of IAHA for a crosslinked product using the Southwestern Ontario database, a big Canadian cohort. For this aim, they selected 1263 patients with OA in 1 or both knees that received 2 consecutive series of IAHA injections, and they compared them to a cohort of 3318 demographically matched KOA patients who were never treated with IAHA. All the evaluations were assessed in a 6-year period between 2006 and 2012. Results showed that in the group of patients who received treatments of IAHA, pain at rest and functional pain after a 6 min walk decreased with an average change of 3.7 ± 1.8 points and 5.6 ± 1.7 points on a 10-point visual analog scale, respectively. At the same time, the distance walked in a 6 min walk test increased on average by 115 m in this patient group. These improvements in pain and physical function were significantly higher than those achieved in KOA-matched patients treated with other medications. Moreover, from the analysis of randomized controlled trials and meta-analyses, an extensive critical literature review conducted in 2018 found a positive effect for the use of IAHA versus placebo with an effect size (ES) of between 0.30 and 0.40 above that of the IA placebo effect [41]. The same review demonstrated that IAHA was as effective as NSAIDs for pain relief and provided a longer-lasting benefit compared to IA corticosteroids from week 8 onwards, with an ES of 0.22 at week 8 rising to 0.39 at week 26 in favor of IAHA. No differences were found comparing a high molecular weight HA formulation to a low molecular weight HA in relieving pain and improving knee joint function, but it was proven that the higher molecular weight caused more burning at the injection site in some patients. Similar evidence is also emerging from studies based on the use of instrumental tests to validate the effectiveness of KOA infiltrative treatments. Moreover, the effects of repeated intra-articular hyaluronic acid on cartilage degeneration using magnetic resonance imaging (MRI) T1ρ mapping were investigated as follows: cartilage degeneration may be improved with a higher number of administrations of IAHA, causing a change in the area of degeneration (*p* < 0.05) independent of age, sex, Kellgren–Lawrence grade, and posterior horn meniscus tears [42]. A significant improvement in cartilage after intra-articular injection with high molecular weight, biological fermentation-derived HYAJOINT Plus was also documented by using ultrasonography (US) in the same population of patients [43]. Similarly, another study showed that patients who received multiple injections of HA had a lower risk of knee replacement surgery compared to patients who did not receive HA injections, thereby also demonstrating the indirect, socio-economic, and long-term benefits of the IAHA [44]. Repeated IAHA injections were strongly recommended as a treatment option in the management of KOA, tailored by disease stage and patient phenotype, and this therapy efficacy was identified in terms of patient-reported outcomes and total knee replacement-sparing effect, remarking the disease-modifying effects of IAHA [40,45]. In this regard, in fact, it should be emphasized that in well-selected cases, the most drastic, though not always completely resolving approach, is the surgical treatment. It has the aim to restore normal knee kinematics and function. Specifically, there are two types of surgical strategies. Unicompartmental knee arthroplasty (UKA) is a bone-conserving and ligament-sparing procedure that can involve the medial or the lateral department of the knee [46]. On the other hand, as understandable from the name, total knee arthroplasty (TKA) consists of both medial and lateral femorotibial joint and patellofemoral joint replacement [47]. Although surgical strategies are effective treatment options, post-operative complications include infection, blood clots, and the loosening or malalignment of prosthetic components, along with the common complications of any surgery in a category of patients who are often elderly and obese. In addition, the choice of prosthesis materials and coatings must also be well-designed. A study conducted in the past year showed that porous tantalum, an orthopedic surgery material widely used for TKA, released debris over time. It appeared that this debris contributed to increased bone absorption and, as a result, caused a catastrophic failure of the tibial baseplate in a 64-year-old physically active Caucasian man with advanced varus right knee [48]. For these reasons, delaying the possibility of surgery as much as possible using IAHA is crucial and, in some cases, even lifesaving. HA act as a senotherapeutic and, precisely, as a senomorphic agent. In fact, it has been shown to attenuate the senescence-associated secretory phenotype (SASP) by upregulating sirtuin 1 (SIRT1) and attenuating oxidative stress. For this reason, it is used in multiple formulations, both topical and systemic, to counteract the evolution of many chronic inflammatory diseases [49,50].

In addition, HA can improve the mitochondrial function and quality control, which are critical for cellular homeostasis and longevity. Furthermore, it promotes the inhibition of matrix metalloproteinase 13 (MMP13), an enzyme that plays a key role in the breakdown of cartilage in OA in general, thus in KOA [36]. MMPs are a family of zinc-dependent endopeptidases that are involved in the degradation of various ECM components, including collagen, aggrecan, and proteoglycans [51]. MMPs are synthesized and secreted by chondrocytes, synoviocytes, and other joint tissues in response to proinflammatory cytokines, such as interleukin-1β (IL-1β) and tumor necrosis factor-α (TNF-α), which are elevated in KOA [52]. MMPs are regulated by various endogenous inhibitors, such as tissue inhibitors of metalloproteinases (TIMPs), but the balance between MMPs and TIMPs is disrupted in KOA, leading to an ECM degradation [51]. MMP13, also known as collagenase 3, is considered a key enzyme in KOA pathogenesis, and its inhibition has been proposed as a therapeutic target [51]. By inhibiting MMP13, HA can help to slow the progression of joint degeneration and preserve joint function. In 2020, a process of temporomandibular joint (TMJ) osteoarthrosis was induced in 36 male albino rats [53]. The goal was to demonstrate how an IAHA treatment could improve the condition of these rats both biologically and functionally. The treated group underwent IAHA injections once a week for a total of three administrations. At the end of the treatment, the treated group showed a regaining of the normal histological features of the TMJ and decreased levels of MMP13 when compared to the non-treated group, thus demonstrating the ability of HA to inhibit collagenase 3 and, consequently, to slow down the articular cartilage degeneration process. From these points of view, therefore, HA can be considered a senomorphic agent, capable of counteracting cellular senescence and slowing down the constant process of osteoarthritis [54,55]. Considering the fundamental role of MMP13 in the pathogenesis of this disease and the great potential of HA in inhibiting this collagenase, a study group created an injectable, self-healing hydrogel from the union of HA and a cell-compatible iron-glutathione (Fe^3+^—GSH) complex in an aqueous environment [56]. The obtained gel demonstrated a higher MMP13 regulation than a commercial HA joint injection and was able to successfully inhibit the synovial fluid MMPs of an osteoarthritic patient. These results induce reflections to new possibilities in the use of HA as both a mechanically competent hydrogel as well as a mediator of MMP regulation for OA therapy in general, thus KOA therapy in particular. In recent years, subchondroplasty, a new “mini-invasive” technique, is gaining popularity and currently appears to have promising results in KOA. It consists of a calcium phosphate injection targeting the osteochondral lesion preceding KOA, also known as bone narrow lesion (BML) [57]. While in an animal model, this technique seems to have optimal results on chondrocytes, unfortunately, recent studies have failed to confirm its chondroprotective effects in humans. In fact, a recent study showed that the use of this technique significantly reduced pain in a 76-year-old patient with resistant medial knee pain, but failed in the long-term, such that the patient underwent TKA 4 years later [58]. Further studies are needed, as it may become another important therapy able to slow down the degenerative progression of KOA and, consequently, reduce the pain and improve joint function in early staged affected patients.

While IAHA injections are generally safe and well-tolerated, there are some potential risks and limitations to consider. Adverse events associated with HA injections are typically mild and transient, such as pain or swelling at the injection site [32]. However, there have been rare cases of more serious adverse events, such as joint infection or allergic reactions. Additional care should be taken in cases where the aim is to combine HA with other drugs, thereby reinforcing their effect but, de facto, increasing the risk of side effects. In addition, the effectiveness of HA injections may vary depending on the severity of KOA and other patient factors, such as age, weight, and comorbidities [59,60]. In this sense, new promising studies are trying to apply the principles of nanomedicine to KOA therapy to enhance and personalize it to the maximum. A novel non-viral vector loaded with growth and differentiation factor-5 (GDF-5) plasmid using chitosan, HA, and chondroitin sulfate was injected into the articular cavities of rabbits and significantly promoted the expression of ECM proteins in chondrocytes, exhibiting good physicochemical properties and low cytotoxicity in slowing down the progression of OA [61].

Kang et al. prepared self-assembled PEGylated ketogenic (PEG/KGN) micelles to create HA hydrogels containing PEG/KGN micelles for OA treatment; this may make it possible to prolong the permanence and articular effects of HA, exploiting the biocompatibility of the micelles that remain inert in biological tissues and are easily associated with many chemical substances. Similarly, other biological vectors are widely experimented to potentiate the effects of hydrophilic and lipophilic drugs traditionally used for treating OA. Liposomes are used for drug delivery because of their high encapsulation capacity and low toxicity [62]; these molecules have been approved by the US Food and Drug Administration as nanocarriers of many NSAIDs, and this allowed a delivery of the drugs to the intra-articular environment avoiding the systemic effects, such as gastric lesions and an increase in blood pressure. The virtuous chemical and biophysical characteristics of HA suggest that it too will soon be associated with liposomes and dendrites, thus overcoming the limitations of topical use of this drug.

Another modern approach is TissueGene C, which is a biological drug of consisting of both cell and gene therapy used in OA treatment, and which comprises two irradiated allogeneic primary chondrocytes and GP2-293 cells. The injection of TissueGene C into the OA knee joint of rabbits determined great benefits in cartilage regeneration and Phase 2 clinical trials have been already approved for moderate knee OA in South Korea [63].

Gene therapy is a promising new treatment strategy for common joint disorders such as OA, but further studies are needed to experiment safety and effectiveness of targeted gene carriers into primary human chondrocytes.

## 5. Conclusions

In conclusion, IAHA injections are a valuable treatment option for patients affected by KOA while they can provide pain relief, improve joint function, and slow the progression of knee joint articular cartilage degeneration. The inhibitory effect of HA on MMP13 and its action as a senomorphic agent suggests that it may have additional benefits beyond its already well-known and documented lubricating and shock-absorbing properties. However, more research is needed to fully understand the mechanisms of action of HA and to optimize its use in clinical practice, also considering the possibility to combining it with other conservative treatments to enhance its beneficial effects.

## Data Availability

Not applicable.
